# Isolation and Evaluation of Bioactive Protein and Peptide from Domestic Animals’ Bone Marrow

**DOI:** 10.3390/molecules23071673

**Published:** 2018-07-09

**Authors:** Parhat Rozi, Palida Maimaiti, Aytursun Abuduwaili, Ahmidin Wali, Abulimiti Yili, Haji Akber Aisa

**Affiliations:** 1 The Key Laboratory of Plant Resources and Chemistry of Arid Zone, Xinjiang Technical Institute of Physics and Chemistry, Chinese Academy of Sciences, No 40-1 Road, Urumqi 830011, China; parhatruzi@126.com (P.R.); Aytursun18@163.com (A.A.); ahmidin@ms.xjb.ac.cn (A.W.); Haji@ms.xjb.ac.cn (H.A.A.); 2 State Key Laboratory Basis of Xinjiang Indigenous Medicinal Plants Resource Utilization, Xinjiang Technical Institute of Physics and Chemistry, Chinese Academy of Sciences, Urumqi 830011, China; 3 University of Chinese Academy of Science, Beijing 100039, China; 4 Department of Nursing, Xinjiang Medical University, Urumqi 830011, China

**Keywords:** bone marrow, protein and peptide, nutritional evaluation, SDS-PAGE, LC/MS

## Abstract

In this work, proteins and peptides were isolated from four kinds of animal bone marrow and characterized by sodium dodecyl sulfate-polyacrylamide gel electrophoresis (SDS-PAGE), Fourier transform infrared spectroscopy (FT-IR), scanning electron microscopy (SEM) and liquid chromatography-mass spectrometry (LC/MS). The antimicrobial and antioxidant activity of these proteins were investigated in vitro. The nutritional value was evaluated by analyzing their free amino acid composition. The results indicates that all of the extracts appeared two bands at SDS-PAGE, the peptide band at 4.1–10 kDa and protein band at 66 kDa, these data are consistent with LC/MS results. FT-IR analysis showed that the secondary structure of protein mainly consists of α-helix. SEM micrographs revealed that the fractions have different morphological characteristics. Horse bone marrow protein (HBMP) showed the highest antioxidant activity to DPPH free radical, IC_50_ value was 0.573 mg/mL. Most of the obtained fractions showed antimicrobial activities towards *Escherichia coli* (EC) and *Candida albicans* (CA). Total free amino acid content ranged between 5.15–49.60 mg/g, and among them, HBMP displayed the highest abundance, 49.7 mg/g, which amino acid composition ratio approached the Food and Agriculture Organization/World Health Organization (FAO/WHO) ideal amino acid pattern recommendation. This study provides fundamental knowledge and a basic study method for the research into and development of animal bone marrow proteins and peptides as functional food and drug resources.

## 1. Introduction

The meat industry annually produces tons of by-products, which results in a huge volume of waste and causes environmental issues [1]. Among these products, livestock bones account for a large proportion. They represent a highly nutritious resource both for food and medicine, which contain a variety of proteins, oils, chondroitin, phosphorproteins, mucopolysaccharides, amino acids, vitamins, calcium, and phosphorus as well as minerals such as iron, zinc, copper, strontium and other health beneficial factors [2,3]. Bone protein, which has numerous biological functions including antioxidant [4], antimicrobial [5], immuno-modulatory [6], antitumor [7] and hypoglycemic [8] activities, is the most important component of animal bone.

Bone is comprised of a protein and calcium network structure, with tubes filled with bone marrow, which is extremely rich in nutrients [9,10]. Previous studies on animal bone have mainly used bone directly for the purpose of manufacturing related products, so there had been no need for the separation of bone and bone marrow, and it consequently resulted in the reduction of the nutritional value of the products on a large scale [11]. Therefore, it is urgent to develop technologies and scientific methods in the field of basic research for the comprehensive utilization of animal bones.

Usually, bone marrow studies are more concerned in bone-related diseases and transplants [12,13]. There is a lack of reports regarding the composition and biological activities of compounds from animal bones, in addition, according to our estimations there have been no reports on the molecular mass and composition of bone marrow. In traditional Chinese medicine, the bones of wildlife species such as tiger, bear, rhinoceros, and sika deer have been the main source of ethnic medicines for the treatment of arthritis [14]. Though more limited in number, ethnic medicine records relating to the bones of domestic animals are increasingly becoming a prime source for modern scientific research and medical applications [15,16]. Yet, research attempts focusing on the properties of bone proteins and peptides as well as their related pharmacological compounds are still missing. Therefore, this study will provide a broad assessment on the discovery and effective utilization of new bioactive monomer peptide resources.

Herein, we intended to provide a technical and theoretical foundation for the development of modern peptide health foods or drugs. Instead of wildlife, our research firstly focused on the isolation and evaluation of bioactive proteins and peptides from the bone marrow of domestic animals. Thus, we narrowed our focus to the bone marrows from sheep (SBMP), bovines (BBMP), horses (HBMP) and camels (CMBP), which were separated from the bone, extracted with water and Tris-HCl buffer, and then fractionated with an ammonium sulfate gradient (30%, 50%, 70%) in order to identify new bioactive proteins, and evaluate the best bone marrow recourses. The isolated fractions were further characterized by SDS-PAGE, FT-IR, SEM and LC/MS. Also, their antimicrobial, and antioxidant activities were assessed in vitro.

## 2. Results and Discussion

### 2.1. Extraction of Bone Marrow Protein (BMP)

As shown in Table 1, the yield of water extracted proteins from different bone marrows was ranked in the order HBMP > SBMP > CBMP > BBMP. Because of its soft texture, more oil content and better water solubility, HBM had the highest extraction yield. The protein content was ranked in the order BBMP > HBMP > CBMP > SBMP. Bovine bone peptide extracted by an enzymatic process has been reported to give the highest yield (69.8%) through using response surface analysis [17]. Our results indicated that bone marrow was an abundant source of proteins and peptides as we obtained the same yield without using any assisted process. The results shows that (Table 2), 50% ammonium sulfate fractionated BBMP and CBMP have the highest protein contents of 52.3 mg/mL and 56.5 mg/mL, respectively. Water extracted BBMP has the highest protein content (52.20 mg/mL) and stronger antimicrobial activity than others, which implies that there has certain dose-dependent relationship between the protein content and antimicrobial activity (Table 3).

Compared with the water-extracted proteins, ammonium sulfate fractionated parts have a higher protein content and antimicrobial activity, which further confirms a dose-dependent relationship between the protein content and antimicrobial activity (Table 3). A study by Zhang [5] revealed that bovine bone collagen hydrolysates obtained with flavourzyme and neutral proteases have antimicrobial activity against *Staphyloccocus aureus* with an inhibition zone diameter of 6.03 mm and 7.97 mm. The results imply that the four kinds of BMP have modestly high antimicrobial activity against CA (Table 1, Table 2 and Table 3).

### 2.2. Nutritional Evaluation

The amino acid composition of proteins plays a vital role in their physiological benefits. The amino acid composition analysis results (Table 4) showed that the water extracted proteins contained 17 kinds of amino acid (Try dissolved in HCl cannot be detected). The total free amino acid content ranged between 5.15–49.60 mg/g, while essential amino/non-essential amino acida (E/N) and (essential amino acid/total amino acid) E/T ranged from 0.61–1.45 mg/g and 0.38–059 mg/g, respectively. Among them, HBMP, SBMP E/T and E/N ratios were close to the FAO/WHO recommended ideal amino acid pattern.

The total free amino acid content ranked in the order HBMP > BBMP > CBMP > SBMP, and the content of drug amino acids was in accordance with the total amino acid order. Comparing the amino acid content, there were different percentages of each free amino acids, where by Gly for the SBMP, Leu for the BBMP, Ser for the HBMP and Ala for the CBMP were the predominant amino acids. BBMP was richer in free amino acids than bone and beef hydrolysate [18,19], which implies that BMP might have a higher nutritional value. Further studies to evaluate the exact amino acid composition and content are recommended.

### 2.3. Characterization of BMP

#### 2.3.1. SDS-PAGE Analysis

**Figure 1:**
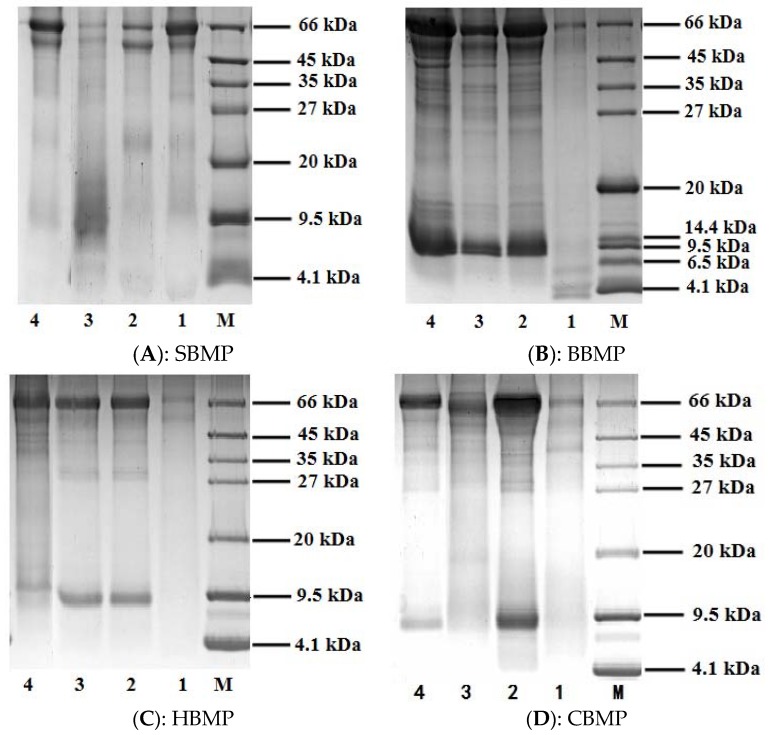
SDS-PAGE (12%) analysis of BMP from four kinds of animals. 1: 30% of ammonium sulphate precipitation part; 2: 50% of ammonium sulphate precipitation part; 3: 70% of ammonium sulphate precipitation part; 4: water extract; M: Marker (4.1–66 kDa).

The protein profiles of the four kinds of animal bone marrow are shown in Figure 1. The protein ladder showed a detection range of 4.1–66 kDa. The results showed that water extracted SBMP, 30% SBMP, HBMP, CBMP and 70% of CBMP ammonium sulfate fractionated parts showed protein bands at 45–66 kDa. The peptide of 30% of BBMP appeared at about 4.1 kDa and the 70% fraction of SBMP appeared at 9.5 kDa. Other parts both appeared at 66 kDa and 9.5 kDa. In conclusion, there are different protein and peptide bands for different bone marrows and they can be differentiated by their protein profiles based on SDS-PAGE analysis. Among them, the main component of 70% fraction of SBMP and 30% of BBMP ammonium sulfate precipitation part were polypeptides, making this part suitable for further separation to find bioactive monomer peptides.

#### 2.3.2. FT-IR Analysis

**Figure 2:**
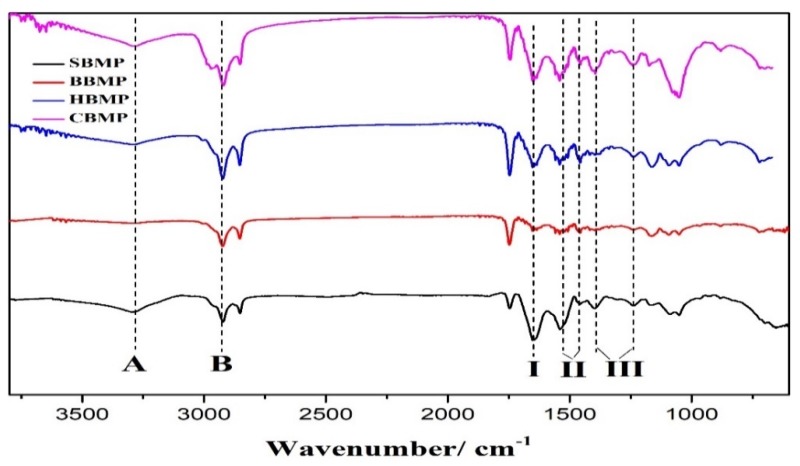
FT-IR spectra of four types of BMP from water extract. (A, B, I, II, III indicate amide A, B, I, II, III band).

The FTIR spectra of the four types of water extracted BMP in the range of 500–4000 cm^−1^ showed characteristic bands of protein compounds (Figure 2). The four kinds of obtained proteins have an absorption peak near 3280 cm^−1^ caused by N–H stretching vibrations, which is the characteristic peak of the amide A band. The absorption peak around 2920 cm^−1^ assigned to the C–N stretching vibration was a typical amide B band peak resulting from the Fermi resonance, and was identified as the overrun of the amide II band. The characteristic peak around 1650 cm^−1^ was for the C=O stretching vibration (amide I band). Two absorption signals between 1450–1600 cm^−1^ were for the amide II band, among them, the peak around 1540 cm^−1^ was assigned to the bending vibration for N–H and the peak at 1460 cm^−1^ was caused by the side chains of amino acid residues from the peptides, which were assigned to N–H vibrations. The amide III band was found around 1200–1400 cm^−1^, and also has two peaks for the C–N bending vibration around 1400 cm^−1^ and the N–H bond around 1240 cm^−1^, respectively. The peak position distribution result are shown in Table 5. The four kinds of BMP were mainly composed of α-helix structures [20,21]. The characteristic absorption peaks of proteins and peptides can be determined by FT-IR analysis, but it cannot explain the main differences between the different proteins. Therefore, further analysis needs to be conducted to evaluate the percentages of each peak.

#### 2.3.3. SEM Analysis

**Figure 3:**
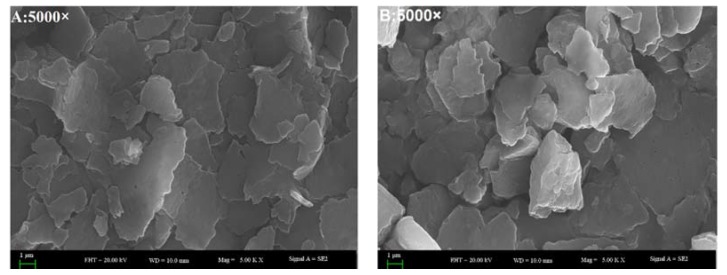

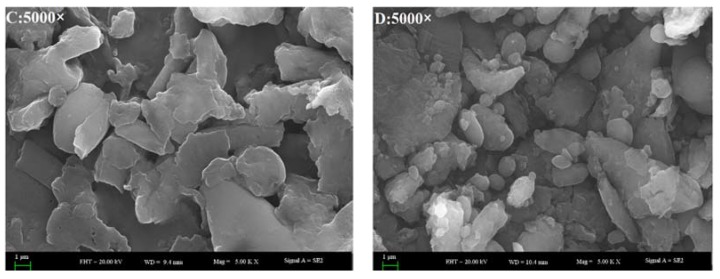
The SEM images of four types of BMP from water extract. (**A**) SBMP; (**B**) BBMP; (**C**) HBMP; (**D**) CBMP.

SEM was used to observe the morphological characteristics of water extracted proteins [22]. The SEM images at a 5000-fold magnification demonstrated that all of the SBMP, BBMP, HBMP and CBMP have characteristic structures (Figure 3). By combining the results with SDS-PAGE, it can be concluded that SBMP was mainly composed of large molecular weight (Mw) proteins. However, the other three parts composed of peptides had low Mw. All of them showed flake-like structures with different sizes, which indicated that the bonding strengths creating aggregations are different. Compared with SBMP, BBMP and HBMP, the surface of CBMP appeared coarser, porous and with more cracks.

#### 2.3.4. Energy-Dispersive X-ray Spectroscopy (EDX) Analysis

**Figure 4:**
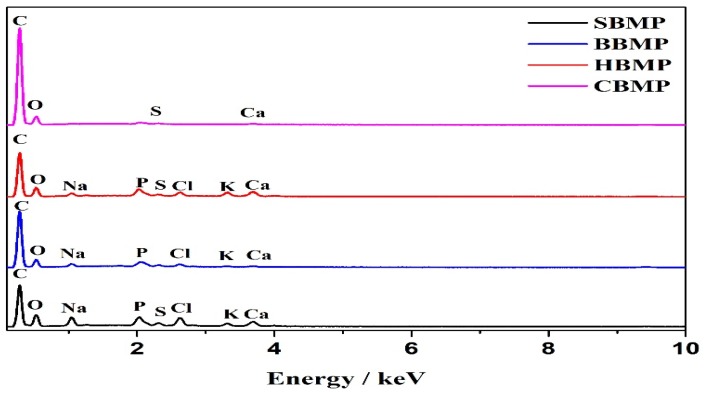
EDX of four kinds BMP.

The elemental analyses of the proteins measured by EDX are shown in Figure 4 and Table 6. The results revealed that SBMP consists 24.29% of O and 70.27% of C, BBMP consists 23.03% of O and 73.09% of C, HBMP consists 19.33% of O and 78.71% of C, CBMP consist 18.10% of O and 81.70% of C, respectively. There were also a few trace elements including K, Ca, Na, P, S and Cl, the HBMP Ca and P content were higher than other parts.

#### 2.3.5. LC/MS Analysis

**Figure 5:**
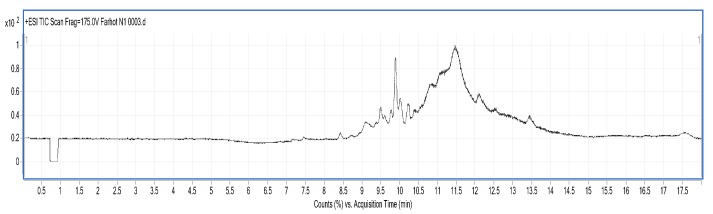
LC-MS chart of SBMP from water extract.

**Figure 6:**
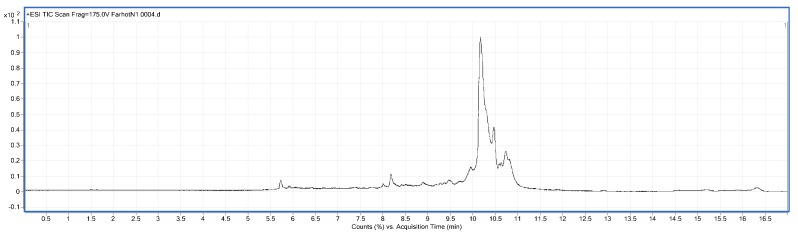
LC-MS chart of BBMP from water extract.

**Figure 7:**
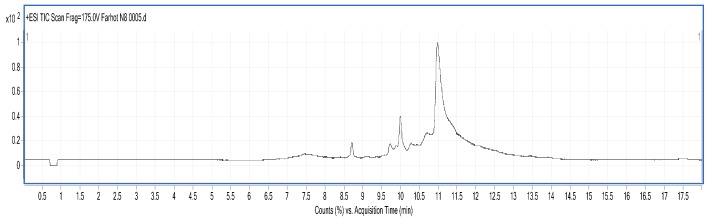
LC-MS chart of CBMP from 50% ammonium sulfate precipitation part.

LC/MS figures and retention time, molecular weight (Mw) information are shown in Figure 5, Figure 6 and Figure 7 and Table 7. The results were consistent with the SDS-PAGE analysis. A total of 28 peptides and nine proteins were found in the water extracted SBMP. The peptide Mw range was 1053.4627–8673.46 Da, and that of the proteins was 10,845.85–18,567.53 Da.

A total of 25 peptides were found in the water extracted BBMP. The Mw of peptides was in the range of 1027.84–9916.32 Da [23]. The maximum charge of peptides is +9, and the corresponding Mw is 4947.51 Da, while the minimum charge is +3, and the corresponding Mw is 2757.38 Da.

A total of nine peptides and two proteins were found in the 50% ammonium sulfate precipitated part of CBMP, where the Mw of the peptides was 1040.35–3986.65 Da, and the Mw of proteins was 11,430.35–15,178.48 Da. The maximum charge of the peptides is +8, and the corresponding Mw 3986.65 Da, while the minimum charge is +4, with a corresponding Mw of 2775.4276 Da.

There are reports showing that net charges evidently contribute to the antimicrobial, antioxidant and anticancer activities of peptides. Wang [24] reported that positively charged peptides contain high contents of basic amino acids, and the hydrophobic interactions between positively charged fractions and the lipid membranes may also contribute to the biological activity. Therefore, it can be concluded that BMP have various potential functions to accelerate biological activities as a relatively large number of charged peptides were detected in three BMP parts. The result might provide a further scientific basis for the effective utilization of natural resources such as BMP and their application in fundamental research.

### 2.4. Antioxidant Activity

**Figure 8:**
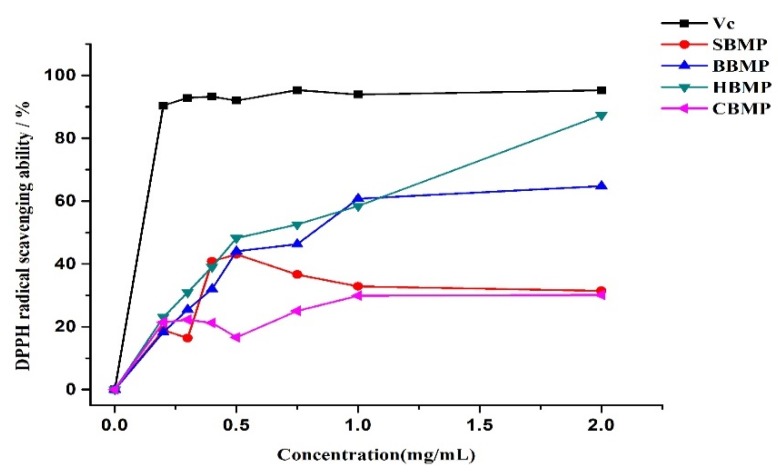
DPPH radical scavenging activity.

The radical scavenging activities of peptides could be influenced by the type, amount and sequence of amino acids, as well as by the degree of hydrolysis and molecular weights of peptides. Antioxidants are beneficial for health as they have strong effects against reactive oxygen species [25,26]. The in-vitro radical scavenging activities of the four kinds of BMP were determined (Figure 8). All of the obtained fractions demonstrated certain scavenging ability against DPPH free radicals within the tested dosage range (0.025–2.0 mg/mL). The scavenging ability of BBMP and HBMP reached a maximum value of 64.7% and 83.9%, respectively, at a concentration of 2 mg/mL, indicating a considerable dose-dependent relationship. The scavenging ability of HBMP was higher than that of other fractions, which might be attribute to the total amino acid content (HBMP: 29.69 mg/g, BBMP: 17.60 mg/g). The IC_50_ values of HBMP and BBMP were 0.573 mg/mL and 0.834 mg/mL, respectively. These results are in accordance with Rajapakse’s results [27], pointing out that aromatic amino acids have a considerable effect against radical scavengers, because they can donate protons easily to electron deficient radicals while at the same time maintaining their stability through resonance structures, which improves the radical-scavenging properties of the amino acid residues.

## 3. Materials and Methods

### 3.1. Materials

Fresh raw materials were obtained from a slaughter house in Urumqi (Xinjiang, China). All of the marrow was removed from the front and rear leg bones, and immediately frozen after rinsing with cold water and warm water three times in order to remove crushed bones and blood, then was crushed using nitrogen (1:6, *w*/*v*) into a powder, which was stored at −20 °C until use. This article does not contain any studies with human participants or animals.

DPPH, bovine serum albumin (BSA), Trizma base, aspartic acid, arginine, glutamic acid, etc. and 18 kinds of amino acid standards were purchased from Sigma Corporation (Cookstown, NJ, USA). The electrophoresis reagentsf, such as 10% SDS, 0.1 mol/L Tris-HCl (pH = 6.8, 8.8) were purchased from Biosharp Corporation (Beijing, china). Running buffer, Bis-Tris gels, and Coomassie Brilliant Blue G 250 were obtained from Invitrogen (Carlsbad, CA, USA). All other chemical reagents used in this study were purchased from local suppliers.

### 3.2. Extraction and Isolation of Bone Marrow Protein (BMP)

For each sample 100 g of bone marrow powder was taken. Firstly extraction was performed with water under the following conditions: solid-liquid ratio 1:5 (*w*/*v*), extraction time 3 h, temperature 45 °C, times 3. The extracts were centrifuged at 10,000 rpm for 10 min and the supernatants were concentrated on a rotary evaporator (BUCHI R300, Flawil, Switzerland), and dialyzed (cut-off 1000 Da) for 48 h against distilled water and lyophilized (FDU-2100, EYELA, Tokyo, Japan). Secondly, samples were extracted with Tris-HCl buffer, and further fractionated with different concentrations of ammonium sulfate (30%, 50%, 70%) for 24 h at 4 °C, then centrifuged at 12,000 rpm for 10 min. The precipitated were washed with deionized water, redissolved, and dialyzed for 48 h. Finally, the solution was lyophilized to obtain a crude BMP powder.

### 3.3. Determination of Protein Concentration and Extraction Yield

The protein concentration of sample was measured by using a Pierce^®^ BCA Protein Assay Kit (Thermo Scientific, Waltham, MA, USA), and expressed as mg/g [28]. Yield of the protein extraction was estimated as follows:

Yield (%) = M_b_ × Ca/M_a_ × Ca × 100% (1)

where M_b_ represents the weight of bone marrow protein (g); M_a_ represents as the weight of bone marrow defatted powder (g) and Ca represents as the protein content (mg/mL).

### 3.4. Sodium Dodecyl Sulfate-Polyacrylamide Gel Electrophoresis (SDS-PAGE)

Electrophoretic analyses of proteins and peptides were carried out according to the reported procedures with slight modifications [29,30] using 12% separating gel and 4% stacking gel. The electrophoresis was run at 75 V for 1 h in the stacking gel and 150 V in the separating gel until the tracking dye reached the bottom of the gel. The gel stored at fixed solution for 1 h and stained with Comassie Brilliant Blue G 250 for 1 h, then held in a decoloring solution for 1.5 h.

### 3.5. Amino Acid Composition of BMP

The amino acid composition was analyzed according to the reported procedures with slight modifications [31]. Four kinds of BMP powder (30 mg) were taken and hydrolyzed with 6 mol/L HCl (10 mL) at 110 °C for 24 h. The hydrolysates were diluted to 50 mL, then 1 mL of solution was evaporated to dryness at 25 °C and diluted to 5 mL with 0.02 mol/L HCl. The phenylisothiocyanate (PITC) method was used to derivatize the samples. The hydrolysates were determined using an amino acid analyzer (Hitachi L-8900, Shimadzu Seisakusho Co., Ltd., Kyoto, Japan).

### 3.6. Fourier-Transform Infrared Spectroscopy Analysis (FT-IR)

The IR spectra were recorde using a Fourier transform infrared spectrophotometer (NICOLET 6700, Thermo fisher, Madison, WI, USA). Samples were pressed into pellets, and then subjected to FT-IR spectrophotometry in the range of 400–4000 cm^−1^.

### 3.7. Scanning Electron Microscope-Energy Dispersive X-ray (SEM-EDX) Analysis

SEM-EDX analysis of the fractions were performed to observe the surface characteristics and elemental analysis. Dried samples of the purified fractions were fixed on a silicon wafer, coated using ion beam sputtering deposition, and the images were collected at a voltage of 20.0 kV with magnification at 2000–10,000× under high vacuum. To determine the elemental composition of the four kinds of extract, an EX-250 energy dispersive X-ray spectrometer (HORIBA Ltd., Kyoto, Japan) equipped with a cold field emission was utilized operating at 5 kV with an analysis time of 50 live seconds.

### 3.8. Identification of BMP by Liquid Chromatography-Mass Spectrometry (LC/MS)

LC/MS analysis was performed using a Series 6520B CHIP-Q-TOF LC-MS instrument (Agilent Technologies, Foster, CA, USA) according to the reported method. The ionization source was ESI+, skimmer cone potential 65 V, drying gas temperature 350 °C, fragmentation at 175 V. Mass range in MS mode 300–3000 *m*/*z*. Positive ionization mode was used. An Agilent Zorbax 300 SB-C18, C18 column (150 mm × 7.5 mm, 5 μm), mobile phases A (0.1% formic acid) and B (MeCN + 0.1% formic acid), gradient procedure: 0–5%/0–3 min, 5–80%/3–13 min, 80%/13–15 min, 5–80%/15–17 min [32,33] were used.

### 3.9. Biological Activity

#### 3.9.1. Antimicrobial Activity

Sample solution (20 μL) was taken from the concentrated 50 mg/mL samples and placed in the incubator at 37 °C for about 30–60 min. A Vernier caliper was used to measure and record the diameter of bacteriostatic halos after 16–18 h. The samples were considers as ineffective when the inhibition zone diameter ≥7 mm. Antimicrobial activity was determined using following microorganisms: CA: *Candida albicans* (ATCC10231), EC: *Escherichia coli* (ATCC11229) [34].

#### 3.9.2. Antioxidant Activity

The DPPH radical scavenging activity of the water extracted proteins was determined. Water extracted protein samples (20 mg) prepared to different concentration (0.025 mg/mL, 0.125 mg/mL, 0.25 mg/mL, 0.5 mg/mL, 0.75 mg/mL, 1 mg/mL, 2 mg/mL) were used. Tests were conducted according to the reported method with some modifications [35]. The DPPH radical scavenging activity was calculated as follows:

Scavenging effect (%) = 1 − (A_i_ − A_j_)/A_0_ (2)

where, A_0_ is the absorbance of the DPPH solution with distilled water; A_i_ is the absorbance of the sample mixed with DPPH solution; A_j_ is the absorbance of the sample with methanol.

## 4. Conclusions

In short, the structural characteristics of four kinds of proteins extracted with water and Tris-HCl buffer were investigated by SDS-PAGE, FT-IR, SEM and LC/MS analysis. Nutritional values were evaluated by determination of the amino acid composition. Biological activities were evaluated by antimicrobial and antioxidant activity in vitro. The properties of SBMP, BBMP, HBMP and CBMP were considerably different, including Mw, morphological structures, amino acid contents and biological activities. The corresponding marrow proteins exhibited significant antibacterial and antioxidant activities, which reveal the potential application of bone marrow protein in functional foods and nutraceuticals. To the best of our knowledge, this is the first report of the in vitro antibacterial and antioxidant activity of peptides derived from sheep, bovine, horse and camel bone marrow proteins.

This research initially verified the potential value of bone marrow proteins as a promising natural bioactive resource. Differences in amino acid sequences between bone marrows will produce different biological effects. Therefore, extensive studies are required for further identification of the peptide sequences responsible for the various biological activities. We insist that animal waste recovery is an important research subject; this work will provide further assistance for the subsequent research on domestic animal bone processing.

## Figures and Tables

**Table 1:** Comparison of water extracted proteins from different bone marrow.

No.	Protein Content (mg/mL)	Extraction Yield (%)
SBMP	20.22	86.85
BBMP	52.20	68.43
HBMP	34.60	90.47
CBMP	25.60	72.07

Notes: SBMP, BBMP, HBMP, CBMP indicate sheep, bovine, horse and camel bone marrow protein.

**Table 2:** Comparison of different BMP by ammonium sulfate fractions.

No.	Concentration	Protein Content (mg/mL)	Extraction Yield (%)
SBMP	30%	40.66	7.95
	50%	22.53	10.79
	70%	25.37	7.38
BBMP	30%	35.11	44.14
	50%	52.35	52.41
	70%	41.71	24.80
HBMP	30%	13.6	55.70
	50%	44.3	11
	70%	25.1	5.5
CBMP	30%	17	15.3
	50%	56.5	7.50
	70%	47.3	4.17

Notes: SBMP, BBMP, HBMP, CBMP indicate sheep, bovine, horse and camel bone marrow protein.

**Table 3:** Comparison of antimicrobial activity from different BMPs.

No.	Concentration	CA (mm)	EC (mm)
water extracted proteins			
SBMP		8	7
BBMP		8	9
HBMP		7	8
CBMP		8	8
ammonium sulfate fractions			
SBMP	30%	9	9
	50%	8	-
	70%	9	-
BBMP	30%	9	9
	50%	10	9
	70%	8	10
HBMP	30%	8	-
	50%	9	8
	70%	7	-
CBMP	30%	8	-
	50%	9	9
	70%	8	-

Notes: CA. *Candida albicans*; EC. *Escherichia coli*.

**Table 4:** The composition and contents of free amino acids in four kinds of water extract BMP.

Amino Acid	SBMP	BBMP	HBMP	CBMP
	Content (mg/g)			
Asp ^d^	0.08	0.12	2.76	0.06
Thr ^e^	0.08	0.29	2.18	0.04
Glu ^d^	0.05	0.25	2.13	0.11
Ser	0.50	3.25	8.83	0.78
Gly ^d^	1.26	1.52	3.21	1.18
Ala	0.53	4.94	6.40	2.15
Val ^e^	0.44	5.97	3.61	1.92
Met ^d^	0.02	1.70	0.33	0.28
Ile ^e^	0.02	1.75	1.30	0.47
Leu ^e,d^	0.10	6.94	4.76	1.71
Tyr ^d^	0.31	3.10	2.39	1.17
Phe ^e,d^	0.18	4.61	3.50	1.14
Lys ^e,d^	1.13	4.21	4.26	1.39
His	0.09	2.43	0.47	0.67
Arg ^d^	0.01	0.12	1.96	0.07
Pro	0.36	1.87	1.54	0.85
Total amino acid (T)	5.15	43.05	49.63	13.98
Essential amino acids (E)	1.97	25.45	19.94	6.95
Non-essential amino acids (N)	3.19	17.60	29.70	7.04
Drug amino acid (D)	3.15	22.57	25.31	7.10
N/T (%)	0.62	0.41	0.60	0.50
E/T (%)	0.38	0.59	0.40	0.50
E/N (%)	0.62	1.45	0.67	0.99
D/T (%)	0.61	0.52	0.51	0.51

Notes: ^e^: essential amino acid, ^d^: drug amino acid.

**Table 5:** Peak positions and assignment of BBM in FT-IR spectra.

Assignment	SBMP	BBMP	HBMP	CBMP	Reason
	Frequency (cm^−1^)				
Amide A band	3282.45	3284.47	3274.05	3282.45	N–H stretching vibration
Amide B band	2923.21	2923.48	2924.20	2920.44	C–N stretching vibration
Amide I band	1647.12	1650.28	1648.48	1652.02	C=O stretching vibration
Amide II band	1539.53	1540.17	1540.04	1540.52	N–H bending vibration
	1460.20	1456.41	1456.54	1464.63	C–N stretching vibration
Amide III band	1399.49	1397.13	1397.72	1398.50	C–N stretching vibration
	1239.42	1238.59	1238.79	1240.55	N–H bending vibration
β-Sheet	-	-	-	-	1616–1637 cm^−1^, 1681–1700 cm^−1^
Random coil	-	-	-	-	1638–1645 cm^−1^
α-Helix	√	√	√	√	1646–1664 cm^−1^
β-Turn	-	-	-	-	1665–1681 cm^−1^

**Table 6:** Results of elemental analysis.

Weight/%	C	O	Na	Cl	Ca	P	K	S
SBMP	70.27	24.29	1.89	1.09	0.88	0.99	0.36	0.31
BBMP	78.71	19.33	0.72	0.31	0.17	0.49	0.11	0.15
HBMP	73.09	23.03	0.80	0.54	0.95	0.96	0.62	-
CBMP	81.70	18.10	-	-	0.12	-	-	0.08

**Table 7:** LC–MS data analysis of four extraction parts.

Sample	Time (min)	Molecular Mass (Da), Charge
Water extracted SBMP	8.437	3916.1385 (+5), 3916.1356 (+4);
	9.074	1190.8024, 1309.4977, 1454.9223, 1636.6824;
	9.369	1571.6103;
	9.509	1071.9013;
	9.607	2196.2814 (+3), 2720.9205 (+3), 1079.2666, 1309.5326, 1473.1616;
	9.764	1055.2924, 1205.9512;
	9.776	4821.01, 6024.33, 8434.93, 10,845.85, 12,029.26, 13,254.79, 15,265.12, 15,902.83, 16,869.74;
	9.879	8673.46, 18,567.53, 16,118.62, 12,207.23;
	9.891	1240.1513, 1446.6508;
	10.018	1273.3925;
	10.244	3387.9831 (+3);
	10.852	1053.4627, 2357.9774;
	11.176	1059.5252, 1756.9654, 2880.9894;
Water extracted BBMP	5.471	1138.7220 (+5), 1215.6066 (+3), 4947.5178 (+9), 4974.0250 (+7), 4972.0210 (+5), 2073.2722;
	5.930	4598.4259 (+7), 4596.64 (+6), 2757.38 (+3);
	5.187–5.634	6589.66, 7487.97, 7787.89; 9916.3219;
	8.916	1044.7616, 1145.2224, 1272.0502, 1422.0194;
	9.491	1138.8300, 1035.7773, 1139.2317;
	10.476	7599.0611;
	10.742	1027.8437, 1116.4654, 1152.2774;
50% part of CBMP	8.689	2776.4385 (+5), 2775.4276 (+4);
	9.720	1040.35, 11,430.35;
	9.991	3986.6528 (+8), 3985.6285 (+7), 3774.3756 (+6), 3773.3610 (+5), 3617.2236 (+6), 3616.2045 (+5);
	10.697	15,178.48;
